# Frequency of Celiac Disease in Pediatric Patients With Chronic Diarrhea

**DOI:** 10.7759/cureus.84428

**Published:** 2025-05-19

**Authors:** Mustafa Kamal, Huma Gul, Abdul Hameed, Shabnam Mahsood, Shehr Bano Raza, Mian Abdur Rehman

**Affiliations:** 1 Pediatrics and Child Health, Hayatabad Medical Complex, Peshawar, PAK; 2 Pediatrics and Child Health, Mercy Teaching Hospital, Peshawar, PAK

**Keywords:** celiac disease, children, chronic, diarrhea, malabsorption, pakistan

## Abstract

Background

Celiac disease (CD) is a prevalent immune-mediated disorder characterized by intestinal mucosal damage in individuals genetically predisposed to gluten sensitivity. The subsequent malabsorption results in gastrointestinal symptoms such as chronic diarrhea and systemic effects like impaired growth and development. Left untreated, CD can lead to various complications, including gastrointestinal malignancies. Therefore, timely diagnosis and management of CD are essential.

Objective

To determine the frequency of celiac disease in children with chronic diarrhea admitted to the Department of Pediatrics, Hayatabad Medical Complex, Peshawar, Pakistan.

Materials and methods

This cross-sectional study was conducted in the Department of Pediatrics, Hayatabad Medical Complex, Peshawar, Pakistan, from January 10, 2024, to March 15, 2025. Children under 14 years of age with a history of chronic diarrhea were enrolled in the study. Children on a gluten-free diet on clinical suspicion without formal testing, those with blood-stained diarrhea, and those with a history of abdominal surgery were excluded. A structured questionnaire was used to collect the following information: age, gender, weight in kilograms, height in centimeters, and history of CD in siblings or parents. Weight for age was categorized into underweight and normal. Similarly, height for age was categorized into short stature or normal stature. All study participants were screened for CD by determining the titers of anti-tissue transglutaminase IgA and IgG antibodies. Data were analyzed by IBM SPSS Statistics for Windows, Version 21 (IBM Corp., Armonk, NY). The presence of CD was stratified across gender, weight-for-age categories, height-for-age categories, and family history of CD. Post-stratification Chi-square test was employed to determine the statistical significance of differences in these variables between those with and without CD. Multivariate logistic regression analysis was carried out to control confounders. A p-value of less than 0.05 was considered significant for all analyses.

Results

Of the 165 patients, CD was diagnosed in 22 (13.3%). Of those with short stature, 15 out of 52 (28.8%) had CD compared to 07 out of 113 (6.2%) patients with normal height for age (p = <0.001). Patients with short stature were 4.4 times more likely than those with normal stature to have CD (95% CI: 1.57 - 12.38; p = 0.005). Compared to patients without a family history of CD, a significantly higher proportion (n =13, 21.3%, versus n = 09, 8.7%) of those with a positive family history were found to have CD (p = 0.021) in univariate analysis. The positive family history of CD lost its significance when adjusted for other risk factors (AOR: 2.25; 95% CI: 0.83 - 6.08; p = 0.109). Age, gender, and weight did not predict CD in these children.

Conclusions

Celiac disease was diagnosed in 22 out of 165 (13.3%) children with chronic diarrhea. Short stature is associated with CD in children with chronic diarrhea. Family history showed a notable but statistically insignificant association with CD. Weight was not found to be associated with CD. It is imperative that pediatricians consider the possibility of CD in patients presenting with chronic diarrhea, particularly in those with short stature.

## Introduction

As infectious disease rates have changed due to improved hygiene, vaccination, and antibiotics, the prevalence of autoimmune diseases has risen globally [1]. Autoimmune diseases result from complex interactions between genetic predisposition and environmental factors such as food, air pollution, infections, stress, and personal lifestyles [2]. Celiac disease (CD) results from exposure of genetically susceptible individuals to gluten, a protein found in wheat and related grains. Nearly all patients have HLA-DQ2 or HLA-DQ8 genes, which make the immune system react against gluten peptides. The immune response leads to small intestinal mucosal injury resulting in villous atrophy, increased intraepithelial lymphocytes, crypt hyperplasia, and malabsorption [3].

Celiac disease is known for its highly variable clinical manifestations. It can present with classical gastrointestinal symptoms, can have extraintestinal manifestations alone or in combination with gastrointestinal symptoms, or may remain asymptomatic [4]. The wide variability in its clinical presentation and, sometimes, the co-existence of other gastrointestinal disorders like irritable bowel syndrome and lactose intolerance make the diagnosis of CD challenging [5]. Chronic diarrhea is one of the most common presentations of CD in children [6]. It reflects malabsorption and, if left untreated, results in nutritional deficiencies, especially iron deficiency anemia, and delayed development in children [7]. The reported frequency of CD in children with chronic diarrhea has varied by region and by the diagnostic criteria used to define chronic diarrhea and CD. Abu-Zekry et al. (2008) from Egypt reported CD in 07 out of 150 (4.7%) children with chronic diarrhea [8]. Similarly, Bhavika et al. (2021) from India in 2021 found 144 out of 890 (16.2%) children with chronic diarrhea as cases of CD [9]. Panezai et al. (2021) screened children presenting with chronic diarrhea and found that 23 out of 188 (12.2%) had CD [10]. In contrast, a study from Bangladesh by Parveen et al. (2018) reported 22 out of 62 (35.5%) children with chronic diarrhea as seropositive for CD, and 19 out of the 22 (86.3%) had biopsy-confirmed disease [11].

Despite the increasing prevalence and recognition of CD globally, there is a lack of recent data regarding the frequency of CD among children with chronic diarrhea in Pakistan. This study aims to provide updated figures on this issue. The results will be valuable for pediatricians and may alter their perspective and clinical suspicion of CD in children with chronic diarrhea. Early diagnosis of these children is crucial to prevent permanent growth retardation and other complications associated with untreated CD. Additionally, this research will pave the way for future studies and ensure that the Pakistani pediatric population is adequately represented in pooled data for systematic reviews and meta-analyses conducted on this topic.

## Materials and methods

This cross-sectional study was approved by the Institutional Review and Ethical Board, Hayatabad Medical Complex, Peshawar (Approval No. 1672, dated December 13, 2023). It was conducted in the Department of Pediatrics, Hayatabad Medical Complex, Peshawar, Pakistan, from January 10, 2024, to March 15, 2025. Children under 14 years of age with a history of chronic diarrhea (defined by the American Gastroenterological Association as passage of three or more liquid stools per day for more than four weeks) were eligible for inclusion in the study [12]. Children on a gluten-free diet on clinical suspicion without formal testing, those with blood-stained diarrhea, and those with a history of abdominal surgery were excluded. Children who met the inclusion and exclusion criteria were enrolled in the study after parental consent. The sample size was 165, considering a 12.2% frequency of CD in children with chronic diarrhea, a 5% margin of error, and a 95% confidence level [10].

A structured questionnaire was used to collect the following information: age, gender, weight in kilograms, height in centimeters, and history of CD in siblings or parents (see Appendices). Weight for age was categorized into underweight (below 5th centile on the Centers for Disease Control growth charts) and normal (above 5th centile on the Centers for Disease Control growth charts) [13]. Similarly, height for age was categorized into short stature (below 5th centile on the Centers for Disease Control growth charts) or normal stature (above 5th centile on the Centers for Disease Control growth charts) [13]. All study participants were screened for CD by determining the titers of anti-tissue transglutaminase IgA and IgG antibodies [14,15]. Celiac disease was diagnosed if the patients were positive for either IgA or IgG antibody. All participants underwent other investigations and treatments tailored to their clinical presentation at the discretion of the attending pediatrician.

Data were analyzed by IBM SPSS Statistics for Windows, Version 21 (IBM Corp., Armonk, NY). Descriptive statistics were calculated for the study variables and presented as median and interquartile range, and frequency and percentage. The median age of patients with and without CD was compared for statistical difference using the Mann-Whitney test. The presence of CD was stratified across gender, weight-for-age categories, height-for-age categories, and family history of CD. Post-stratification Chi-square test was employed to determine the statistical significance of differences in these variables between those with and without CD. Multivariate logistic regression analysis was carried out to control confounders. A p-value of less than 0.05 was considered significant for all analyses.

## Results

Of the 165, most patients were male (n=107, 64.8%). The median age of the study participants was seven years (IQR: 5 - 9 years). Most (n=118, 71.5%) were underweight for their age. Celiac disease was diagnosed in 22 (13.3%) patients. Table 1 summarizes the characteristics of the study population.

**Table 1:** Characteristics of the study participants (n = 165) * Data for age represented as Median (IQR), while data for gender, weight for age, height for age, family history of celiac disease, and celiac disease represented as No. (%) IQR: interquartile range

Variables	Median (IQR) No. (%)*
Age in years, Median (IQR)	7 (5 - 9)
Gender, No. (%)	
Male	107 (64.8%)
Female	58 (35.2%)
Weight for age, No. (%)	
Underweight	118 (71.5%)
Normal	47 (28.5%)
Height for age, No. (%)	
Short stature	52 (31.5%)
Normal stature	113 (68.5%)
Family history of celiac disease, No. (%)	
Positive	61 (37%)
Negative	104 (63%)
Celiac disease, No. (%)	
Yes	22 (13.3%)
No	143 (86.7%)

The median age of patients with and without CD was not significantly different (6 years (IQR: 4 - 9) versus 7 years (IQR: 5 - 9), respectively, p = 0.578). Similarly, gender was not associated with the diagnosis of CD (p = 0.277). More patients (n = 19,16.1%) in the underweight-for-age group had CD than those with normal weight for age (n = 03, 6.4%), but the difference did not reach statistical significance (p = 0.097). Short stature was found to have a significant association with the diagnosis of CD. Of those with short stature, 15 out of 52 (28.8%) had CD compared to 07 out of 113 (6.2%) patients with normal height for age (p = <0.001). Compared to patients without a family history of CD, a significantly higher proportion (n =13, 21.3% versus n = 09, 8.7%) of those with a positive family history were found to have CD (p = 0.021). Table 2 summarizes the results of univariate analysis of risk factors for CD.

**Table 2:** Relationship between various risk factors and celiac disease in children with chronic diarrhea (n = 165) @ Data for age represented as Median (IQR), while data for gender, weight for age, height for age, and family history of celiac disease represented as No. (%) ^ Mann-Whitney U value for age, and chi-square value for gender, weight for age, height for age, and family history of celiac disease $ P-value of less than 0.05 considered statistically significant * Mann-Whitney test # Chi-square test IQR: interquartile range

Variables	Celiac disease^@^		Test statistic^	P value^$^
	Yes	No		
Age in years, Median (IQR)	6 (4 – 9)	7 (5 - 9)	1458.0	0.578*
Gender, No. (%)				
Male	12 (11.2)	95 (88.8%)	1.182	0.277^#^
Female	10 (17.2%)	48 (82.8%)		
Weight for age, No. (%)				
Underweight	19 (16.1%)	99 (83.9%)	2.747	0.097^#^
Normal	03 (6.4%)	44 (93.6%)		
Height for age, No. (%)				
Short stature	15 (28.8%)	37 (71.2%)	15.812	<0.001^#^
Normal stature	07 (6.2%)	106 (93.8%)		
Family history of celiac disease, No. (%)				
Positive	13 (21.3%)	48 (78.7%)	5.331	0.021^#^
Negative	09 (8.7%)	95 (91.3%)		

Multivariate regression analysis revealed that patients with short stature were 4.4 times more likely than those with normal stature to have CD (95% CI: 1.57 - 12.38; p = 0.005). Though associated with the diagnosis of CD in univariate analysis, the positive family history of CD lost its significance when adjusted for other risk factors (AOR: 2.25; 95% CI: 0.83 - 6.08; p = 0.109). Gender and weight for age did not show a significant association with CD in multivariate analysis (Table 3).

**Table 3:** Multivariate logistic regression analysis of risk factors for the presence of celiac disease # P-value of less than 0.05 considered statistically significant * Reference category AOR: adjusted odds ratio; CI: confidence interval

Variables		AOR	95% CI	P-value^#^
Gender	Female*			
	Male	0.56	0.21 – 1.51	0.252
Weight for age	Normal*			
	Underweight	1.96	0.50 – 7.64	0.333
Height for age	Normal stature*			
	Short stature	4.41	1.57 – 12.38	0.005
Family history of celiac disease	Negative*			
	Positive	2.25	0.83 – 6.08	0.109

## Discussion

Celiac disease is a condition caused by immune-mediated damage to the intestinal mucosa due to gluten exposure in genetically susceptible children. Left untreated, CD can lead to significant intestinal and extraintestinal complications, including malignancies, as well as adverse effects on children's growth and quality of life [4]. Maintaining a high index of suspicion for diagnosing and managing CD in children is important to prevent negative outcomes. Malabsorption resulting in chronic diarrhea is one of the most common manifestations of CD. This study aimed to determine the frequency of CD in the pediatric population with chronic diarrhea.

Of the 165, CD was diagnosed in 22 (13.3%) patients. The frequency of CD in children with chronic diarrhea has varied across different studies. Panezai et al. (2021) have reported CD in 23 out of 188 (12.2%) children with chronic diarrhea [10]. Likewise, Bhavika et al. (2021) from India have reported CD in 144 out of 890 (16.2%) children with chronic diarrhea [9]. In contrast, Abu-Zekry et al. (2008) from Egypt and Parveen et al. (2018) from Bangladesh have reported CD in 07 out of 150 (4.7%) and 22 out of 62 (35.5%) children with chronic diarrhea, respectively [8,11]. These variations reflect the differences in the inclusion criteria among the studies, specifically the operational definition used for chronic diarrhea. For example, the sample studied by Abu-Zekry et al. (2008) also included patients whose main presentation was failure to thrive [8]. Similarly, the cut-off duration to define "chronic" diarrhea has ranged from two weeks to three months in previous studies [9,10].

The median age of children diagnosed with CD was six years (IQR: 4 - 9). This is consistent with the findings of Poddar et al. (2006) from India (mean age 6.7 ± 3 years) [16]. In other studies, the mean age at diagnosis has ranged from 5.5 to 9.5 years [17-19]. However, Canadian children have been diagnosed at the median age of three years [20]. The younger age at diagnosis might be attributed to increased screening of children and the availability of screening facilities in developed countries. Over the years, the median age at diagnosis of CD has increased, with a decrease in the severity of symptoms at presentation [21]. In a study focused on the clinical presentation of Finnish children with CD, Kivelä et al. (2015) have reported that the median age at the time of diagnosis has increased from 4.3 years in the 1980s to 7.6 and 9.0 years in the last two decades [22]. The subtle nature of the symptoms may contribute to delays in diagnosis. Additionally, certain patients exhibit solely extraintestinal manifestations, which may not prompt the pediatrician to consider the possibility of CD early on in these cases.

The frequency of CD in this study was not affected by the patient's gender. Systematic reviews and meta-analyses have reported that the prevalence of CD is higher in girls than in boys, similar to other autoimmune conditions [23,24]. The absence of such an association in this study may be due to the comparatively smaller sample size relative to population-based studies and systematic reviews, as well as the lower representation of girls within our sample.

In comparison to patients with normal weight for age, a greater proportion of underweight patients were diagnosed with CD in this study; however, the difference was not statistically significant. Historically, being underweight has been considered indicative of undiagnosed CD [25]. Recent studies have questioned this theory, showing that a notable number of children diagnosed with CD are overweight or obese [26,27]. Weight alone is not a reliable predictor of CD, as most cases still fall within the normal weight range [28]. This issue introduces a novel challenge in the diagnosis of CD in pediatric patients. Based on the findings from these studies, CD should be considered in children of normal weight as well as those who are overweight or obese, especially in cases where there is a family history of autoimmune disorders or extraintestinal manifestations of the disease.

Short stature was found to have a significant association with undiagnosed CD in children experiencing chronic diarrhea, and this relation remained statistically significant in multivariate analysis. This association has been consistently documented in previous studies. Muhammad et al. (2022) reported CD in 51 out of 152 (33.7%) children with short stature [29]. Short stature can be the only presenting complaint of children with undiagnosed CD, and early introduction of a gluten-free diet can result in catch-up growth [30].

This study observed that children with a family history of CD were diagnosed with CD at a significantly higher rate. However, this association lost statistical significance after adjustment for other variables in a multivariate analysis. Genetic predisposition is a recognized risk factor for the development of CD in children, often manifesting at an earlier age [31]. Due to the prevalence of extraintestinal manifestations and asymptomatic disease in adults, combined with the absence of registries for patients with genetic disorders and the limited availability of genetic screening for families with index cases of CD, it is challenging to reliably collect the history of familial predisposition in resource-constrained environments. Research involving larger sample sizes is necessary to better elucidate this association.

The primary strength of this study lies in its emphasis on children from a region where CD, despite its prevalence, is often diagnosed late. The application of multivariate analysis to control for confounding variables ensures the study's internal validity. Nonetheless, certain limitations should be acknowledged. The study's single-center design, relatively small sample size, and exclusive reliance on serological markers without histological confirmation are notable shortcomings. These factors may limit the generalizability of the findings and may also have contributed to the failure to identify some well-established associations with CD, such as familial predisposition.

## Conclusions

Celiac disease continues to be a significant concern in pediatric patients experiencing chronic diarrhea. Pediatricians should consider CD, especially in children with chronic diarrhea and short stature, irrespective of their weight status. The absence of a family history of CD should not exclude this diagnosis from the differentials of chronic diarrhea. Maintaining a high level of clinical suspicion for early diagnosis and the prompt initiation of a gluten-free diet can prevent complications associated with untreated CD. Further multicenter studies with larger sample sizes are recommended to overcome the limitations of single-center research and to identify predictors of celiac disease more effectively.

## Appendices

**Table 4:** Frequency of celiac disease in pediatric patients with chronic diarrhea (data collection tool/questionnaire) MR: medical record; kg: kilogram; cm: centimeter; anti-TTG: anti-tissue transglutaminase

S. No.	Variables	Data/questions/response
1	Date	
2	MR number	
3	Age	Years
4	Weight	kg
5	Height	cm
6	Gender	Male/female
7	Weight for age	Underweight/normal weight
8	Stature	Short/normal
9	Family history of celiac disease	Yes/no
10	Anti-TTG antibodies	Positive/negative

## References

[1] Miller FW (2023). The increasing prevalence of autoimmunity and autoimmune diseases: an urgent call to action for improved understanding, diagnosis, treatment, and prevention. Curr Opin Immunol.

[2] Pisetsky DS (2023). Pathogenesis of autoimmune disease. Nat Rev Nephrol.

[3] Iversen R, Sollid LM (2023). The immunobiology and pathogenesis of celiac disease. Annu Rev Pathol.

[4] Adams DW, Moleski S, Jossen J, Tye-Din JA (2024). Clinical presentation and spectrum of gluten symptomatology in celiac disease. Gastroenterology.

[5] Rossi RE, Masoni B, Zullo A, De Deo D, Hassan C, Repici A (2024). Clinical presentation of celiac disease in adult patients: current real-life experience. Intern Emerg Med.

[6] Guandalini S, Assiri A (2014). Celiac disease: a review. JAMA Pediatr.

[7] Sahin Y (2021). Celiac disease in children: a review of the literature. World J Clin Pediatr.

[8] Abu-Zekry M, Kryszak D, Diab M, Catassi C, Fasano A (2008). Prevalence of celiac disease in Egyptian children disputes the east-west agriculture-dependent spread of the disease. J Pediatr Gastroenterol Nutr.

[9] Bhavika YM, Prasanna Kumar DG, Harish HN (2021). Celiac disease in children with chronic diarrhoea: a cross-sectional study. J Clin Diagnostic Res.

[10] Panezai MS, Ullah A, Ballur K (2021). Frequency of celiac disease in patients with chronic diarrhea. Cureus.

[11] Parveen S, Karim AB, Rahman SM, Alam MR, Ahmed DS, Rahman MZ (2018). Celiac disease in children with chronic diarrhoea attending at paediatric gastroenterology and nutrition department of BSMMU. Mymensingh Med J.

[12] (1999). American Gastroenterological Association medical position statement: guidelines for the evaluation and management of chronic diarrhea. Gastroenterology.

[13] (2025). CDC growth charts. https://www.cdc.gov/growthcharts/cdc-charts.htm.

[14] Gillett HR, Freeman HJ (2000). Comparison of IgA endomysium antibody and IgA tissue transglutaminase antibody in celiac disease. Can J Gastroenterol.

[15] Korponay-Szabó IR, Dahlbom I, Laurila K (2003). Elevation of IgG antibodies against tissue transglutaminase as a diagnostic tool for coeliac disease in selective IgA deficiency. Gut.

[16] Poddar U, Thapa BR, Singh K (2006). Clinical features of celiac disease in Indian children: are they different from the West?. J Pediatr Gastroenterol Nutr.

[17] Ramosaj-Morina A, Keka-Sylaj A, Zejnullahu AB (2020). Celiac disease in Kosovar Albanian children: evaluation of clinical features and diagnosis. Curr Pediatr Rev.

[18] Pooni PA, Chhina RS, Jaina BK, Singh D, Gautam A (2006). Clinical and anthropometric profile of children with celiac disease in Punjab (North India). J Trop Pediatr.

[19] Tanpowpong P, Broder-Fingert S, Katz AJ, Camargo CA Jr (2012). Age-related patterns in clinical presentations and gluten-related issues among children and adolescents with celiac disease. Clin Transl Gastroenterol.

[20] Rashid M, Cranney A, Zarkadas M (2005). Celiac disease: evaluation of the diagnosis and dietary compliance in Canadian children. Pediatrics.

[21] Tapsas D, Hollén E, Stenhammar L, Fälth-Magnusson K (2016). The clinical presentation of coeliac disease in 1030 Swedish children: Changing features over the past four decades. Dig Liver Dis.

[22] Kivelä L, Kaukinen K, Lähdeaho ML (2015). Presentation of celiac disease in Finnish children is no longer changing: a 50-year perspective. J Pediatr.

[23] El-Metwally A, Toivola P, AlAhmary K (2020). The epidemiology of celiac disease in the general population and high-risk groups in Arab countries: a systematic review. Biomed Res Int.

[24] King JA, Jeong J, Underwood FE (2020). Incidence of celiac disease is increasing over time: a systematic review and meta-analysis. Am J Gastroenterol.

[25] Olén O, Montgomery SM, Marcus C, Ekbom A, Ludvigsson JF (2009). Coeliac disease and body mass index: a study of two Swedish general population-based registers. Scand J Gastroenterol.

[26] De Giuseppe R, Bergomas F, Loperfido F, Giampieri F, Preatoni G, Calcaterra V, Cena H (2024). Could celiac disease and overweight/obesity coexist in school-aged children and adolescents? a systematic review. Child Obes.

[27] Diamanti A, Capriati T, Basso MS (2014). Celiac disease and overweight in children: an update. Nutrients.

[28] van der Pals M, Myléus A, Norström F (2014). Body mass index is not a reliable tool in predicting celiac disease in children. BMC Pediatr.

[29] Muhammad A 2nd, Arif N, Wajid KK, Rehman K, Sardar N, Khan P, Hussain U (2022). Short stature and celiac disease in children (5 to 16 Years) presenting at a tertiary care hospital in Peshawar. Cureus.

[30] Meazza C, Pagani S, Gertosio C, Bozzola E, Bozzola M (2014). Celiac disease and short stature in children. Expert Rev Endocrinol Metab.

[31] Meijer CR, Auricchio R, Putter H (2022). Prediction models for celiac disease development in children from high-risk families: data from the PreventCD cohort. Gastroenterology.

